# Facilitators and Barriers of Smokers’ Compliance with Smoking Bans in Public Places: A Systematic Review of Quantitative and Qualitative Literature

**DOI:** 10.3390/ijerph13121228

**Published:** 2016-12-11

**Authors:** Li Zhou, Lu Niu, Hui Jiang, Caixiao Jiang, Shuiyuan Xiao

**Affiliations:** 1 Department of Social Medicine and Health Management, School of Public Health, Central South University, Changsha 410078, China; zhouli97328@126.com (L.Z.); angela_niulu@hotmail.com (L.N.); jh1985126@126.com (H.J.); 2 Centre for Health Behaviors Research, JC School of Public Health and Primary Care, The Chinese University of Hong Kong, Hong Kong, China; 3 Department of Epidemiology and Health Statistics, School of Public Health, Central South University, Changsha 410078, China; xiaoxiaocx123@126.com

**Keywords:** facilitators, barriers, smoking bans, public places, environmental tobacco smoke

## Abstract

*Background*: Environmental tobacco smoke (ETS) exposure is associated with an increased risk of many diseases. Many countries have ratified a national smoking ban in public places, but studies on factors related to smoking issues in public places post-ban are lacking. *Aim*: To identify facilitators and barriers that influenced smokers’ compliance with smoking bans in public places. *Methods*: Using PubMed, MEDLINE, and the Web of Science database, we conducted a systematic search of English articles published before June 2015 on factors of smokers’ compliance with the smoking bans in public places. *Results*: A total of 390 references were identified, among which seventeen articles (twelve quantitative studies, two qualitative studies, three mixed-method studies) were included in this review. These studies focused on four types of public places including recreational venues (*n* = 7), hospital (*n* = 5), school (*n* = 4), and workplace (*n* = 1). Factors at the  individual-, interpersonal-, and organizational-level were identified: at the individual level, nicotine dependence, insufficiency of tobacco-related knowledge, and the negative attitudes towards smoking bans were the most commonly identified barriers; at the interpersonal level, the smoking behaviors of people around, close relatives, and friends’ approval were the main barriers; and at the organizational level, the main barriers were inefficient implementation of the bans and the inconvenience of the designative smoking areas. *Conclusions*: This synthesis of the literature provided evidence of the identified barriers and facilitators of smokers’ compliance with the smoking bans. It will be beneficial for the policy-maker to consider interventions on multiple levels of factors to overcome the barriers and enhance smokers’ compliance with the smoking bans in public places.

## 1. Introduction

Exposure to environmental tobacco smoke (ETS) has been associated with premature death and morbidity [1]; it has been documented to be as harmful as active smoking and is the cause of a wide spectrum of morbidity and more than 600,000 premature deaths worldwide [2]. There is no safe level for ETS exposure [3] and, globally, there are more than one billion smokers who can potentially expose others to ETS [4]. In response to this important public health issue, as of September 2010, national smoke-free legislation, which is a key policy under the WHO Framework Convention on Tobacco Control (FCTC), has been ratified by over 170 countries [5]. The main goals of a smoking ban in public places are to protect non-smokers from the dangers of environmental tobacco smoke and provide a supportive environment for those who want to quit smoking [6,7]. It has been well documented that smoking bans are beneficial for improving cardiovascular health outcomes and reducing smoking-related mortality [8]. Different countries have different attitudes towards the smoking bans and strategies for effective implementation. The bans differ across countries [9]. Studies have shown that developed countries were more successful in implementing the smoking bans than developing countries [10] where the support for the bans might be limited by the lack of knowledge and awareness about the adverse effects of passive smoking [11]. A recent review also found the impact of legislative bans on smoking prevalence and tobacco consumption [12]. Smokers’ individual characteristics may affect whether smokers choose to smoke in public places post-ban [5]. For example, some smokers were indifferent to both their own health and others’ [13] and viewed smoking as their freedom and right, so the health promotion on the banning smoking in the public places might be less effective for them [14]. It is also difficult for some smokers to voluntarily comply with the ban without the external restrictions, such as the responsible, sufficient monitoring and effective legal enforcement system [15]. Even though some smokers realize the harm of environmental tobacco smoke [16], the appearance of nicotine withdrawal symptoms and cigarette cravings can make smokers violate the ban unintentionally [17,18]. 

In order to promote the implementation of smoking bans in the public places and deal with the issue of continued smoking post-ban, it is imperative to explore the factors related to the smokers’ compliance with the bans [19]. To date, no such overview has been provided. Therefore, this review aims to fill the gap and identify the facilitators and barriers of smokers’ compliance with smoking bans in the public places.

## 2. Methods

### 2.1. Search Strategy

A comprehensive search was conducted using PubMed, MEDLINE, and the Web of Science database for all studies published before June 2015 on the facilitators and barriers of smokers’ compliance with smoking bans in public places. We used a set of combinations of keywords in the literature search, including words reflecting smokers (extended to patients, employees, students, staff, and patrons), compliance (e.g., comply, compliant), smoke-free (e.g., nonsmoking, no-smoking), ban (e.g., legislation, rule), predictor (e.g., barrier, factor), and public places (e.g., school, hospital). The full search strategy with adapted terms is included in supplementary material.

### 2.2. Inclusion and Exclusion Criteria

Studies included should provide either qualitative, quantitative or mixed-method (i.e., cross-sectional, case-control, and cohort design) data on barriers and/or facilitators of smokers’ compliance with the smoking bans in the public places and been written in English. Studies that focused on places, such as home, personal car, outdoor, and prison, were excluded. Reviews, comments, letters, posters, book chapters, and books were also excluded. Two reviewers (Zhou L. and Jiang C.) independently conducted the screening by title and abstract firstly and then further reviewed the full texts for eligibility. Wherever differences occurred between the two reviewers, consensus was reached by mutual discussion.

### 2.3. Data Extraction

Data about the included studies were extracted, which included the name of the first author, year of publication, study setting (the type of public places and country), study design, sampling methods, sample characteristics, barriers and/or facilitators of smokers’ compliance with the smoking bans in the public places, and measurements/definitions of compliance.

### 2.4. Data Synthesis

All themes relevant to facilitators and barriers of smokers’ compliance with smoking bans in public places were identified and extracted from all eligible studies. Through discussion and consensus among three reviewers (Zhou L., Niu L., and Jiang H.), these themes were summarized and then categorized using the following main domains of the Social Ecological Model [20,21]: individual level, interpersonal level, and organizational level.

### 2.5. Quality Assessment

The quality of the included studies was assessed using the quality assessment tool “QUALSYST” from the study “Standard Quality Assessment Criteria for Evaluating Primary Research Papers from a Variety of Fields” [22]. It contains 14 items for quantitative study and 10 items for qualitative study. Each item was scored based on the degree to which the specific criteria were met (“yes”  =  2, “partial”  =  1, and “no”  =  0). Items which were not applicable (marked as “NA”) were excluded from the summary score. The summary score for each study was calculated as the division of the total score obtained across relevant items by the possible maximum scores. Mixed-method studies were assessed by quantitative and qualitative studies items separately, and then the average score was calculated as a summary score. The summary score ranged from 0 to 1 with a higher score indicating better quality. Two reviewers (Zhou L. and Jiang C.) independently evaluated the quality of the included articles. Wherever the ratings differed between the two reviewers, they discussed until a consensus was reached.

## 3. Results

### 3.1. Search Results

**Figure 1:**
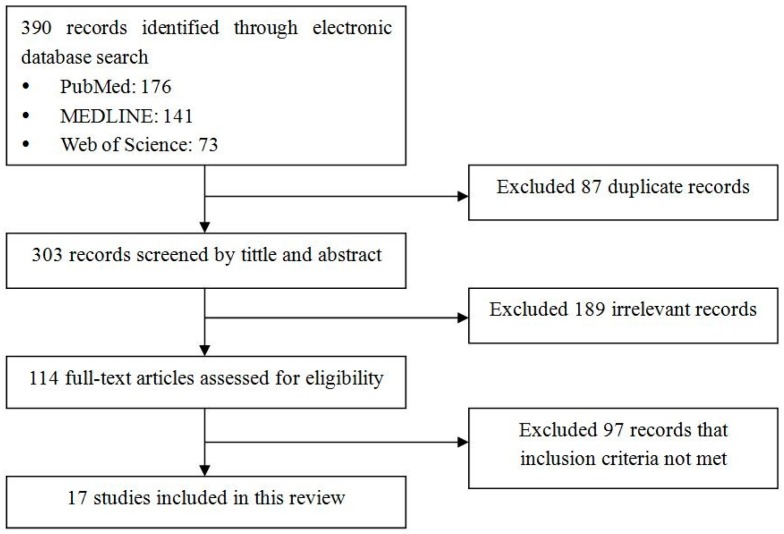
Flowchart for review of studies on facilitators and barriers of smokers’ compliance with smoking bans in public places.

The flowchart of literature search is presented in Figure 1. A total of 390 articles were identified in the search, and 87 duplicated articles were retrieved from three databases. After the evaluation of titles and abstracts, 189 irrelevant articles were excluded. We assessed 114 full-text articles for eligibility and, finally, 17 articles published between 1998 and 2015 were included in this review. In these included studies, one article [23] was published before 2000, eight articles [16,17,18,24,25,26,27,28] were published between 2000 and 2009, and eight articles [5,9,29,30,31,32,33,34] were published after 2010. 

### 3.2. Characteristics of the Study

As shown in Table 1, the included studies focused on four types of public places including recreational venues (e.g., bars, restaurants; *n* = 7) [5,9,24,26,27,28,29], hospital (*n* = 5) [17,18,23,25,30], school (*n* = 4) [16,31,32,33], and workplace(*n* = 1) [34]. Eight studies were conducted in North America (two in Canada [26,30] and six in the USA [18,23,24,28,29,33], four in European countries [16,17,25,31], one in Asian countries [9], one in Australia [32], and three in multiple countries [5,27,34]. Twelve quantitative studies [5,9,16,17,18,23,25,26,27,29,31,34] were identified in this review. Nine studies were cross-sectional [9,16,17,23,25,26,27,31,34] and three were cohort [5,18,29]. Sample sizes ranged from 101 to 9046 participants. In the survey instrument, six studies [18,23,27,29,31,34] used self-designed questionnaires, six studies [5,9,16,17,25,26] used the scale from the published literature, five studies [9,17,23,25,26] of which provided information on the reliability or validity. Two qualitative studies [30,32] used semi-structured interviews and purposive sampling methods, with a sample size of 37 [32] and 82 [30] respectively. Additionally, three studies used mixed-method design [24,28,33].

### 3.3. Risk of Bias

Overall, the quality of the included studies was good. Breakdown of quality appraisal markings was shown in the supplementary materials (Tables S1 and S2). The QualSyst scores ranged from 0.72 [28,33] to 0.95 [29], with a median score of 0.85 and an interquartile range of 0.77–0.91. For 15 quantitative studies (including three mixed method studies), the most frequently missing one was control of potential confounders. For five qualitative studies (including three mixed-method studies), where study quality was diminished was lack of verification procedure.

### 3.4. Barriers and Facilitators

#### 3.4.1. Individual Level

As shown in Table 2, people who were heavier smokers [9,16,18,25,29,31], had heavier nicotine dependence [18,24,26,30], had more severe nicotine withdrawal symptoms [17,18], and had illicit drug consumption [31] were more likely to be noncompliant with the smoking bans in public places. In addition, smokers who had no quitting attempts [18] or were in the earlier stage of quitting and with less confidence on successful quitting [17] were more likely to smoke in public places in defiance of the bans. Three studies each reported supportive attitudes towards smoking [9,32,34] and negative attitudes towards the smoking bans [9,32,33]. Having lower education levels [31] and lower levels of knowledge of smoking harms [16] or being unaware of policy boundaries [32,33] were also reported as barriers of compliance with the bans. For hospitalized smokers with physical mobility limitations, it was inconvenient to smoke outside of the hospital [30].

In terms of facilitators, having high levels of knowledge about the harms of smoking and passive smoking [5,9,34] and supportive attitudes towards the smoking bans [5,27,33] were the most commonly reported facilitators of compliance with smoking bans in public places. Smokers who were lighter smokers, without substance abuse, had confidence to quit smoking [23] and those having negative attitudes towards smoking [5] were more likely to be compliant with the smoking bans. People with a history of heart disease and recent dyspnea also tended to be compliant with the bans.

Smokers’ socio-demographic profiles were also associated with their compliance with the bans, but results were mixed. Two studies reported that being older was the facilitator [18,23], while one identified it as a barrier [31]. Additionally, one study indicated that male smokers were more likely to be compliant with the bans than female smokers [17].

#### 3.4.2. Interpersonal Level

Two studies identified that peers’ dissuasion [33] and no parental permission [31] were facilitators. Meanwhile, the smoking status of peers [9,16] and with the approval from close relatives and friends [16] were barriers. People smoking in the same public setting were also identified as a barrier [24,28,29].

#### 3.4.3. Organizational Level

Efficient implementation of the smoking bans in public places, such as enacting documented smoking bans [27] and formulating rewards and punishment measures [30,33], could effectively decrease smoking in public places. However, in the places that lack surveillance, smokers would be more likely to be noncompliant with the bans [30]. Two studies indicated that the presence of ashtrays in public places was associated with more smokers’ smoking post-ban [24,28]. In a study conducted in bars, there were more customers smoking with only female bartenders on duty, for females had less ability or willingness to enforce the bans [24,28].

In places without convenient designative smoking areas, smokers would be more likely to smoke in no-smoking areas when they needed to [30,33]. Studies suggested that a designated smoking place with an apparent sign [30], convenient traffic, and safe circumstances [30,32,33] were associated with less smoking in public places. Features of the public places were also reported to be related to smokers’ compliance. A study in San Francisco found higher smoking rates in bars with Asian and Irish patrons predominantly than those with Latino patrons predominantly [24,28]. Additionally, a study in American campuses suggested that smokers from public schools were less compliant than those from private schools, especially religious schools [31].

## 4. Discussion

This review brings together studies on the barriers and facilitators of smokers’ compliance with the smoking bans in public places. Although research on this topic remains underdeveloped by including both quantitative and qualitative studies, this review identified a range of factors at the individual-, interpersonal-, and organizational-levels that tobacco researchers, controllers, and policy-makers should consider. 

At the individual level, one of the important factors we identified was knowledge. People who were better informed with the harms of smoking and passive smoking and aware of the policy boundaries were more likely to be compliant with the smoking bans in public places than those with a lower level of knowledge [5,9,16,27,32,33,34]. Additionally, the negative attitudes towards smoking cessation and smoking bans are also identified as one of the key barriers of the smokers’ compliance with the smoking bans [5,9,27,32,33,34]. These results emphasize the need for tobacco-related education for the public and the need to increase publicity of the smoking bans.

Another key barrier was nicotine dependence, i.e., heavy smokers who had heavier nicotine dependence and more severe nicotine withdrawal symptoms were more likely to smoke in the public places regardless of the bans [9,16,17,18,24,25,26,29,30,31]. This implies that smoking cessation programs are needed to help heavy smokers deal with the issue of nicotine dependence, and it needs both higher levels (e.g., interpersonal- and organizational-levels) and individual-level of intervention to deal with their noncompliance with the smoking bans.

At the interpersonal level, according the result, not getting the approval from parents or friends is an important facilitator to the smokers’ compliance with the smoking bans [31,33]. Meanwhile, smoking status of peers [9,16] and with the approval from close relatives and friends [20] were the barriers. This indicates that group interventions could be effective on decreasing smoking issues in the public places post-bans, as the impacts of parents, close relatives, and friends on smokers’ smoking behavior are considerable. Additionally, the people around smoking in the same public setting was the barrier [24,28,29]. One of the reasons for this phenomenon is the lack of explicit enforcement of the bans [35].

In organizational level, implementation of smoking bans is identified as an important factor of compliance [24,27,28,30,33]. The compliance can be strengthened through documented bans, strict measures, and powerful surveillance. Provision of ashtrays could be regarded as tacit approval of smoking in public places [24,28], which weaken the enforcement of smoking bans. These results indicate that each measure of implementing smoking bans should be taken into account to improve smokers’ compliance. To deal with this problem, many interventions are needed, including legislation on smoke-free public places that document the responsibilities of the owners and managers (e.g., the owner of a bar or restaurant, and the director of a hospital), surveillance, corresponding condemnatory regulation, and penalties [14].

Another significant factor identified is the convenience of smoking area [30,32,33]. There is no doubt that smoking and environmental tobacco smoke are harmful for health. Thus, no smoking in public places is needed for the health of smokers and non-smokers [36]. We must protect the nonsmokers’ right to health, but we cannot ignore and deprive of the smokers’ right to make their own choices [37]. In addition, as mentioned before, smoking could be physiologically needed sometimes because of the appearance of nicotine dependence or nicotine withdrawal symptoms [17,18,26,32]. This makes compulsory abstinence a painful challenge for smokers [30], particularly for those who cannot leave a non-smoking area for extended periods of time, such as people with physical mobility limitations and hospitalized patients [30]. Therefore, smokers’ rights and their physiological and psychological smoking needs should be considered when implementing smoking bans. Setting convenient smoking area and prompting them to smoke in designated areas will be helpful to improve smokers’ compliance with the bans.

Several studies pointed that compliance with smoking bans differed in different places, and similarity of compliance exists in different groups [9,24,27,28,31]. Although no culture-related variables are identified in the existing literature, the impacts of social norms and culture on the smokers’ compliance with the smoking bans should be considered. For example, there is a Chinese saying that “a cigarette builds a bridge, while wine builds a road” [38]. Cigarette sharing is a common social practice in China [14,39,40], and it may influence their openness to smoke inside recreational settings even if there is a smoking ban [8]. In addition to the three levels of factors discussed above, future studies should explore and address the barriers and facilitators related to social norms and culture. In order to improve the implementation of the smoking bans in public place, the policy-makers and implementers need to make interventions and strategies targeting barriers at multiple levels.

## 5. Strengths and Limitations

In this study, three levels of barriers and facilitators of smokers’ compliance with smoking bans in public places were summarized. Although we used the most relevant databases (i.e., PubMed MEDLINE, and the Web of Science database), gray literature and some literature in other databases might be missed. Additionally, there is a possible selection bias due to included only English papers and, therefore, might have ruled out some relevant literature in other languages reflecting the impact of culture. Despite these limitations, the findings of this study will be beneficial for the policy-makers and public health researchers to take tailored measures to enhance smokers’ compliance according to these identified factors.

## 6. Conclusions

**T**his synthesis of the literature provided evidence of the identified barriers and facilitators of smokers’ compliance with smoking bans in public places at individual-, interpersonal-, and organizational-levels. At individual level, interventions targeting smoking-related knowledge and attitudes towards smoking cessation and smoking bans should be prioritized. At the interpersonal level, researchers and interveners could plan group interventions, as the impact of close family members, relatives, and friends on smokers’ smoking behaviors are considerable. At the organizational level, the priority should be stricter enforcement of the existing bans and improvement of the convenience of designative smoking areas. More studies are needed to explore and address the barriers and facilitators related to social norms and culture. It will be beneficial for the policy-makers, tobacco researchers, and controllers to consider interventions on the multiple levels of factors to enhance smokers’ compliance with smoking bans in public places.

## Figures and Tables

**Table 1:** Overview of included studies.

First Author and Year	Risk of Bias	Setting/Country	Study Design	Sampling Methods	Sample Characteristics (Sample Size, Gender, Age)	Measurement/Definition of Compliance or Non-Compliance
Quantitative study						
Rigotti, 2000 [18]	0.86	Hospital/U.S.	Cohort	Random	650 inpatient smokers; 55% male; age: 49.2 ± 16.2 years	Patients who did not smoke while hospitalized or smoked outdoors only were classified as compliant; those who reported smoking indoors were noncompliant.
Sabidó, 2006 [17]	0.91	Hospital/Spain	Cross-sectional	Convenient	229 inpatient smokers; 77% male; age: 50 ± 16.9 years	Compliant: those who did not smoke indoors or who only smoked outdoors; noncompliant: those who smoked indoors.
Parks, 2009 [25]	0.77	Hospital/UK	Cross-sectional	Convenient	101 smoking staff; 22.8% male	Those who are compliant with smoke-free policy and only ever smoke off the site; those who are non-compliant and continue to smoke on site.
Lazuras, 2009 [16]	0.73	University/Greek	Cross-sectional	Convenient	182 undergraduate smokers	Whether they had ever smoked in a smoke-free sector in public settings.
Lazuras, 2012 [34]	0.73	Companies/Greece and Bulgaria	Cross-sectional	Random	170 daily or weekly smokers	Compliance with smoking restrictions in smoker-free sectors at work
Galán, 2012 [31]	0.91	Schools/Spain	Cross-sectional	Cluster	1116 student smokers; 42.0% male	Having smoked sometime in the last thirty days on school premises in open or closed spaces.
Emmons, 1998 [23]	0.77	Hospitals/U.S.	Cross-sectional	Convenient	358 hospitalized smokers; 45% male; mean age: 46 years	Adherence was defined as self-reporting of abstaining from cigarettes during the hospital stay.
Lacchetti, 2001 [26]	0.86	restaurants,workplaces, bingo halls, and hockey arenas/Canada	Cross-sectional	Random	423 adult smokers	Compliance with more restrictions.
Li, 2010 [9]	0.86	Recreational venues/China	Cross-sectional	Stratified multistage cluster sampling	2403 smokers who reported patronizing recreational venues; 84.0% males; age: 47.36 ± 8.53	Smoking vs not smoking in recreational settings.
Nagelhout, 2011 [5]	0.91	Bars/Ireland, France, Netherlands, Germany	Cohort	Probability sampling	4634 smokers;	Smoking in smoke-free bars.
Irvin, 2015 [29]	0.91	Korean bars and restaurants/U.S.	Cohort	Probability sampling	224 current smokers of Korean descent who visited a Korean bar or restaurant bars and restaurants; 84.4% male;	Smoked inside Korean bars or restaurants.
Borland, 2006 [27]	0.95	recreational venues/U.S., Canada, UK, and Australia	Cross-sectional	Stratified random sampling	9046 adult smokers; female (52.7%–56.6%)	Smoking inside recreational venues.
Qualitative study						
Shopik, 2012 [30]	0.85	Hospital/Canada	Semi-structured interview	Convenient	82 current smokers	Smoking in the hospital during hospitalization.
Jancey, 2014 [32]	0.80	University/Australian	An environmental audit; direct observation; intercept interview.	Convenient	37 smokers (27% staff and 73% students); 83.8% male; 59.4% aged between 18 and 29 years	Smoking behavior on campus.
Mixed-method study						
Moore, 2006 [28]	0.72	Bars/U.S.	Structure observations, semi-structure interviewers	Study 1: random Study 2: opportunistic sampling	Study 1: 479 observations study 2: 35 bar staff and patrons	Non-compliance: patron smoking.
Moore, 2009 [24]	0.78	Bars/U.S.	High-structure naturalistic observations, semi-structured interviews	Random	121 stand-alone bars	Indoor smoking by bar patrons and staff.
Russette, 2014 [33]	0.72	School/U.S.	Semi-structure interview with 22-item survey and two open-ended questions	Convenient	60 student and non-student smokers; 52% male; mean age: 28 years	Smoking on campus property or off campus property.

**Table 2:** Facilitators and barriers to smokers’ compliance with smoking bans in public places.

Level	Facilitators	Studies	Barriers	Studies
Individual level	Demographic factors			
	Male	1 [17]	Low education level	1 [31]
	Being older	2 [18,23]	Being older	1 [34]
	Smoking behaviors			
	Lighter smokers	1 [23]	Heavier smokers	6 [9,16,18,25,29,31]
			Heavier nicotine dependence	4 [17,18,26,30]
			Nicotine withdrawal symptoms	2[17,18]
	Quiting smoking			
	Having confidence toquit smoking	1 [23]	No confidence to quit smoking	1 [17]
			No quit smoking attempts	1 [18]
			Earlier stage of quit smoking	1 [17]
	Without substance abuse	1 [23]	Illicit drug consumption	1 [31]
	Higher level of knowledge about smoking and passive smoking	3 [5,9,34]	Less awareness of harms of smoking	1 [16]
	Negative attitudes towards smoking	1 [5]	Supportive attitudes towards smoking	3 [9,32,34]
	Supportive attitudes towards the bans	3 [5,27,33]	Negative attitude towards smoking	3 [9,32,33]
			Unawareness of policy boundaries	2 [32,33]
	History of chronic dieases (e.g., dyspnea, heartdisease)	1 [18]	Limited physical mobility	1 [30]
Interpersonal level	No parental permission	1 [31]	Smoking behaviors of people around in the same setting	3 [24,28,29]
			Smoking status of the peers	2 [9,16]
	Peers’dissuasion	1 [33]	Close relatives and friends’ approval	1 [16]
Organizational level	Efficient implementation	3 [27,30,33]	Lack of surveillance	3 [24,28,30]
	Convenience of the designative smoking area	2 [30,33]	Inconvenience of the designative smoking area	3 [30,32,33]
	Private schools (e.g., religious schools)	1 [31]	Only female bartenders were on duty	1 [24]
			Bars serving predominantly Asian or Irish patrons	2 [24,28]

## Supplementary Materials

The following are available online at www.mdpi.com/1660-4601/13/12/1228/s1, Search strategy, Quantitative studies, Qualitative studies, Table S1: Breakdown of quality appraisal markings for 14 articles reporting on studies using quantitative methods, Table S2: Breakdown of quality appraisal markings for 10 articles reporting on studies using qualitative methods.
