# Supplementary material for: Facilitators and Barriers of Smokers’ Compliance with Smoking Bans in Public Places: A Systematic Review of Quantitative and Qualitative Literature
Source: Int J Environ Res Public Health. 2016 Dec 11;13(12):1228. doi: 10.3390/ijerph13121228 (PMC5201369; doi:10.3390/ijerph13121228)
Supplement: Supplementary file 1 [file ijerph-13-01228-s001.pdf]

Supplementary Materials: Facilitators and Barriers of Smokers' Compliance with Smoking Bans in Public Places: A Systematic Review of Quantitative and Qualitative Literature

Li Zhou, Lu Niu, Hui Jiang, Caixiao Jiang and Shuiyuan Xiao

1. Search Strategy

1 compliance/
2 comply/
3 compliant/
4 adhere*/
5 Or/1–4
6 smoke-free/
7 nonsmoking/
8 no-smoking/
9 smok*/
10 Or/6–9
11 rule*/
12 legislation*/
13 bans/
14 restriction*/
15 law/
16 laws/
17 policy/
18 ordinance/
19 Or/11–18
20 predictor*/
21 determinant*/
22 barrier*/
23 facilitat*/
24 enable*/
25 reasons/
26 failure/
27 success/
28 factor*/
29 Characteristics/
30 variables/
31 Or/20–30
32 public places/
33 school/
34 hospital/
35 bar/
36 restaurants/
37 recreational venues/
38 Pubs/
39 campus/
40 worksites/
41 public and service sectors/
42 hospitality venues/
43 Stores/
44 workplaces/
45 Leisure-Hospitality Sector/
46 Or/32–45
47 Patients/
48 employees/
49 students/
50 smokers/
51 staff/
52 patrons/
53 Or/47–52
54 5 AND 10 AND 19 AND 31 AND 46 AND 53

2. Table S1

Table S1. Breakdown of quality appraisal markings for 14 articles reporting on studies using quantitative methods [1].

Study	Item on Kmet et al. Checklist														Summary Score
	1	2	3	4	5	6	7	8	9	10	11	12	13	14	
Rigott et al., 2000	2	2	2	2	NA	NA	NA	1	2	2	2	1	2	1	19/22 = 0.86
Sabidó et al., 2006	2	2	2	1	NA	NA	NA	1	2	2	2	2	2	2	20/22 = 0.91
Parks et al., 2009	2	2	1	1	NA	NA	NA	1	2	2	2	0	2	2	17/22 = 0.77
Lazuras et al., 2009	2	2	1	1	NA	NA	NA	2	1	2	2	0	1	2	16/22 = 0.73
Lazuras et al., 2012	2	2	1	1	NA	NA	NA	2	1	2	2	0	1	2	16/22 = 0.73
Galán et al., 2012	2	2	1	1	NA	NA	NA	2	2	2	2	2	2	2	20/22 = 0.91
Emmons et al., 1998	2	2	1	1	NA	NA	NA	2	1	2	2	0	2	2	17/22 = 0.77
Lacchetti et al., 2001	2	2	2	1	NA	NA	NA	2	1	2	2	2	1	2	19/22 = 0.86
Li et al., 2010	2	2	1	1	NA	NA	NA	2	2	2	2	1	2	2	19/22 = 0.86

Nagelhout et al., 2011	2	2	2	2	NA	NA	NA	1	2	2	2	1	2	2	20/22 = 0.91
Irvin et al., 2015	2	2	1	1	NA	NA	NA	2	2	2	2	2	2	2	20/22 = 0.91
Borland et al., 2006	2	2	2	1	NA	NA	NA	2	2	2	2	2	2	2	21/22 = 0.95
Moore et al., 2006	2	2	1	1	NA	NA	NA	1	1	2	2	0	1	2	15/22 = 0.68
Moore et al., 2009	2	2	1	1	NA	NA	NA	2	2	2	2	0	2	2	18/22 = 0.82
Russette et al., 2014	2	2	1	1	NA	NA	NA	1	1	2	2	0	1	2	15/22 = 0.68

The quantitative scale consists of fourteen items with scores from zero to two and the possibility to score "not applicable" ("not applicable" items were excluded from the calculation of the summary score). The maximum total score is 28. The summary score was calculated by summing up the total obtained scores across the relevant items and dividing that by the total possible score (i.e., 28—number of "not applicable"× 2).

3. Table S2

Table S2. Breakdown of quality appraisal markings for 10 articles reporting on studies using qualitative methods [1].

Study	Item on Kmet et al. Checklist										Summary Score
	1	2	3	4	5	6	7	8	9	10	
Shopik et al., 2012	2	2	2	2	2	2	2	0	2	1	17/20 = 0.85
Jancey et al., 2014	2	2	2	2	1	2	2	0	2	1	16/20 = 0.80
Moore et al., 2006	2	2	2	1	1	2	2	0	2	1	15/20 = 0.75
Moore et al., 2009	2	2	2	1	1	2	2	0	2	1	15/20 = 0.75
Russette et al., 2014	2	2	2	1	1	2	2	0	2	1	15/20 = 0.75

The qualitive scale consists of ten items with scores from zero to two and the possibility to score "not applicable" ("not applicable" items were excluded from the calculation of the summary score). The maximum total score is 20. The summary score was calculated by summing up the total obtained scores across the relevant items and dividing that by the total possible score (i.e., 20—number of "not applicable"× 2).

4. Quantitative Studies

(1) Question or objective sufficiently described?
(2) Design evidence appropriate to answer the study question?
(3) Method of subject selection (and comparison group selection, if applicable) or source of information/input variables (e.g., for decision analysis) is described and appropriate.
(4) Subject (and comparison group, if applicable) characteristics or input variables/information (e.g., for decision analyses) sufficiently described?
(5) If random allocation to treatment group was possible, is it described?
(6) If interventional and blinding of investigators to intervention was possible, is it reported?
(7) If interventional and blinding of subjects to intervention was possible, is it reported?
(8) Outcome and (if applicable) exposure measure(s) well defined and robust to measurement/misclassification bias? Means of assessment reported?
(9) Sample size appropriate?
(10) Analysis described and appropriate?
(11) Some estimate of variance (e.g., confidence intervals, standard errors) is reported for the main results/outcomes (i.e., those directly addressing the study question/objective upon which the conclusions are based)?
(12) Controlled for confounding variables?
(13) Results reported in sufficient detail?
(14) Do the results support the conclusions?

5. Qualitative Studies

(1) Question/objective clearly described?
(2) Design evidence appropriate to answer the study question?

(3) Context for study is clear?
(4) Connection to a theoretical framework/wider body of knowledge?
(5) Sampling strategy described, relevant, and justified?
(6) Data collection methods clearly described and systematic?
(7) Data analysis clearly described, complete, and systematic?
(8) Use of verification procedure(s) to establish credibility of the study?
(9) Conclusions supported by the results?
(10) Reflexivity of the account?

Reference

1. Kmet, L.M.; Lee, R.C. *Standard Quality Assessment Criteria for Evaluating Primary Research Papers from a Variety of Fields*; Institute of Health Economics: Edmonton, AB, Canada, 2004.

© 2016 by the authors. Submitted for possible open access publication under the terms and conditions of the Creative Commons Attribution (CC-BY) license (http://creativecommons.org/licenses/by/4.0/).
